# Preparation and Properties of Polyester Modified Waterborne High Hydroxyl Content and High Solid Content Polyacrylate Emulsion

**DOI:** 10.3390/polym11040636

**Published:** 2019-04-08

**Authors:** Zhewen Zhu, Chaoying Zhang, Shuling Gong

**Affiliations:** College of Chemistry and Molecular Sciences, Wuhan University, Wuhan 430072, Hubei, China; zhuzhewen@whu.edu.cn (Z.Z.); whu-chem-zcy@163.com (C.Z.)

**Keywords:** waterborne coatings, hydroxyl polyacrylate emulsion, polyester modification, seeded semi-continuous emulsion polymerization

## Abstract

A high hydroxyl content waterborne polyester-acrylate emulsion was successfully synthesized in two steps. Firstly, the carboxyl terminated unsaturated polyester was synthesized, then it was reacted as a monomer with acrylate monomer by emulsion polymerization using the semi-continuous seeded method. The effects of the amount of hydroxyethyl methacrylate (HEMA), the ratio of polyester/acrylic, the ratio of soft/hard monomer, and the content of chain transfer agent to the properties of the composite emulsion were investigated. Through a variety of tests, both the emulsion and film properties of the composite emulsion were better than polyacrylate emulsion. The introduction of polyester improved the flexibility and impact resistance of hydroxyl acrylate film, and made the modified resin have advantages of both.

## 1. Introduction

Coatings are widely used in people’s lives. Organic solvents in solvent-based coatings are harmful. With the improvement of people’s requirements for environmental protection, research on water-based coatings has been developed [1,2].

Polyacrylate resin has the advantage of light color, good gloss and color retention, high hardness, water resistance, corrosion resistance and UV resistance, which are widely used in furniture, metal, plastic, leather, paper, building and textile finishing [3,4]. The main factor for the rapid development of water-based acrylic resin is its outstanding performance, but it also inevitably has some disadvantages, mainly reflecting its heat-adhesiveness and cold brittleness after resin drying, and heat resistance, which is not good [5,6]. In order to overcome these limitations, functional monomers or other resins need to be introduced into the acrylate resin for modification [7,8].

Among them, hydroxyl acrylate has attracted much attention and become a research hotspot due to its advantages such as active functional groups, good light and color retention, and high hardness [9,10], especially in the field of automotive coatings where it has been widely used. Acrylates containing hydroxyl functional monomers can be cross-linked with epoxy resins or amino resins by chemical reactions between the hydroxyl group and the epoxy group or amino group [11,12]. It can also be used as a hydroxyl component in waterborne two-component polyurethane coatings to curing with polyisocyanate by crosslinking [13,14]. Thus, the crosslinking density of the film-forming material and the performance of the coating can be improved. Because the content of polar groups such as carboxyl and hydroxyl in the aqueous dispersion of hydroxyl acrylic resin was too high, it is easy to cause the abnormal viscosity of the system in the process of aqueous dispersion [15]. This abnormal viscosity is usually reduced by adding a high boiling solvent to a prepolymer or by increasing the amount of the solvent or by directly reducing the content of the hydroxyl group. Therefore, at present, many products have some defects, such as low hydroxyl value, low solid content, high viscosity, high VOC content and insufficient crosslinking density, which leads to an unsatisfactory coating resistance [16]. Zhang [17] used 2-hydroxyethyl methacrylate as a monomer to provide a high content of hydroxyl group. Waterborne polyacrylate was prepared through a seeded semi-continuous emulsion polymerization method with a pre-emulsification process. Through the optimization of polymerization conditions and the control of the structure of particles, they successfully synthesized the core-shell structure of polyacrylate emulsion with a good appearance, low viscosity and a solid content of 46.5%. The hydroxyl polyacrylate exhibited good performance in the emulsion, but the mechanical properties of the film still needed to be improved.

Polyester resin has some advantages such as good luster, high fullness, an easy to achieve high solid differentiation, and acrylic resin pollution resistance, good adhesion, light and color characteristics, so polyester modified acrylate has become a new research direction [18,19]. Schork et al. [20] synthesized unsaturated polyester modified acrylic resin by a hybridization microemulsion polymerization, which was made into paint film with uniform luster. But under this condition, the result was a polymer with a high gel content. A hybrid system can be obtained by grafting acrylic monomers in situ onto unsaturated polyester molecular chains. Therefore, this resin after hybridization not only has the advantage of unsaturated resin, but also reflects the good performance of acrylic resin.

**Figure 1:**
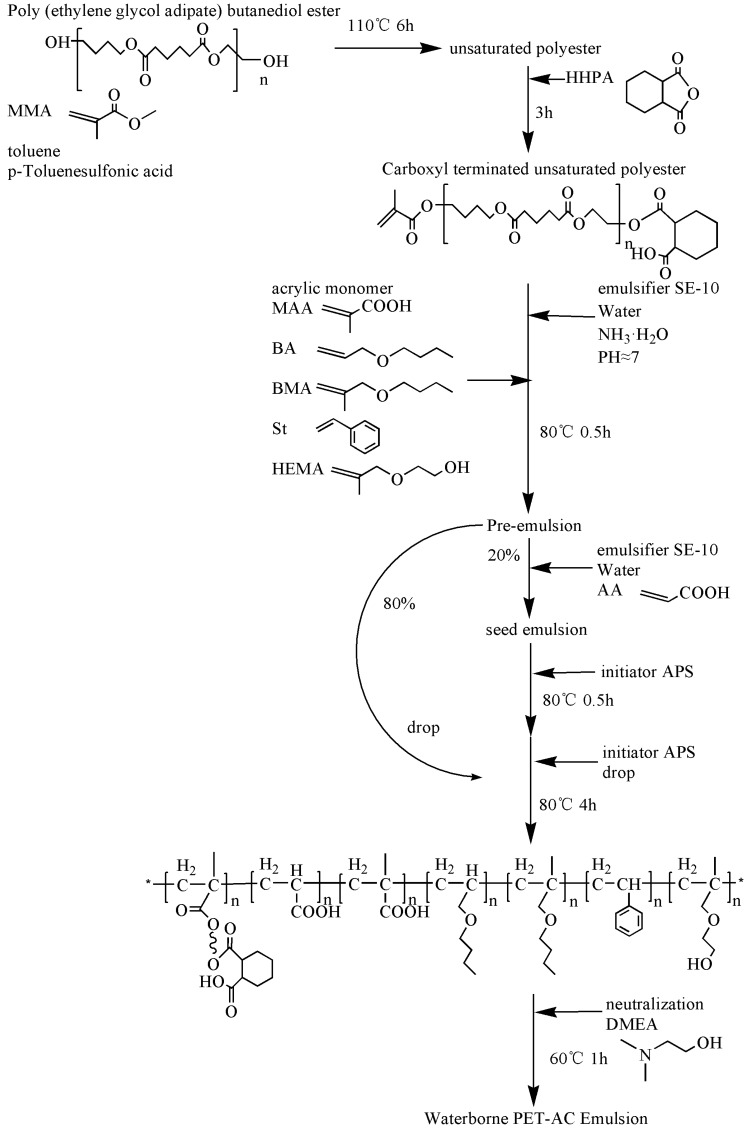
Synthetic route of waterborne PET-AC emulsion.

In this paper, the carboxyl-unsaturated polyester was synthesized first, and was chemically modified as a monomer in acrylate copolymerization. A pre-emulsification and seeded semi-continuous emulsion polymerization method was employed to synthesize the hydroxyl polyester-acrylate composite emulsion (Figure 1). The optimum amount of HEMA in the synthesis of modified resin and its effect on emulsion were investigated. The changes in viscosity, molecular weight and particle size distribution of modified resin emulsion after polyester introduction were investigated. The ratio of polyester/acrylic, the ratio of soft/hard monomers and the amount of chain transfer agent were studied on the emulsion properties, glass transition temperature and thermal stability of the resin.

## 2. Materials and Methods

### 2.1. Materials

Methyl methacrylate (MMA), toluene, p-toluenesulfonic acid, cis-1,2-cyclohexanedicarboxylic anhydride (HHPA), acylic acid (AA), methacrylic acid (MAA), butyl acrylate (BA), n-butyl methacrylate (BMA), styrene (St), hydroxyethyl methacrylate (HEMA), 1-dodecanethiol, ammonium persulfate (APS), ammoniumhydroxide (NH_3_·H_2_O), *N*,*N*-dimethylethanolamine (DMEA), were provided by Sinopharm Chemical Reagent Co., Ltd. (SCRC, Shanghai, China). Reactive emulsifier SE-10 was purchased from Foshan Kodi Gas Chemical Industry Co., Ltd (Foshan, China). Poly(ethylene buthlene glycol adipate) ester were provided by Zhongshan Daoqum Co., Ltd (Zhongshan, China), the molecular weight of Poly(ethylene buthlene glycol adipate) ester was 1000. Dimethylolpropionic acid (DMPA) was purchased from Shanghai Aladdin Co., Ltd (Shanghai, China). In order to eliminate the inhibitor, the activated carbon adsorption method and alkali washing with NaOH or NaHCO_3_ was used for the water-soluble monomer (AA, MAA, HEMA) and other monomer (BA, BMA, St), respectively. Other materials were used without further purification.

### 2.2. Methods

**Preparation of Unsaturated Polyester.** An appropriate amount of Poly(ethylene buthlene glycol adipate) ester and MMA were added in the reaction flask, while toluene was used as a solvent and p-toluenesulfonic acid as the catalyst were added, too. Additionally, the temperature was raised to 110 °C. After about 6 h of reaction, a vacuum device was connected to remove methanol, and pumping was stopped until there was no more distillate. Cis-hexahydrophthalic anhydride was added to capping after about 3 h and then the solvent was removed by pumping until the system viscosity increased and no bubbles were visible. The temperature was lowered to 80 °C and the carboxyl terminated unsaturated polyester (PET) was prepared in the reaction flask for use.

**The preparation of polyester modified acrylate emulsion.** MAA, BA, BMA, HEMA, St, 1-dodecanethiol were added into the three-necked flask to be mixed with PET. After mixing, the temperature was raised to 80 °C, SE-10 and water were mixed and added into the flask for pre-emulsification for 0.5 h. Ammonia was added to adjust the pH ≈ 7, after that, 80% pre-emulsion was removed with the constant pressure dropping funnel. AA was added into the flask and mixed with pre-emulsion, 20 wt % of the APS was dissolved in water and added to the flask, and the remaining 80% pre-emulsion and initiator were dripped slowly. The reaction was continued for 4 h after completion of the dropwise addition. During the reaction, the amount of initiator was added according to the degree of reaction of the monomer and the viscosity of the resin, and the temperature was raised to 90 °C at the later stage of the polymerization. After that, the mixture was cooled to 60 °C, and the neutralizing agent DMEA was added to neutralize the emulsion. Then, the reaction temperature was lowered to room temperature. The PET-AC composite emulsion was prepared.

The neutralization degree of the whole reaction was 0.9. The content of the emulsifier SE-10 and the initiator APS were 1.5% and 2%, respectively.

### 2.3. Characterizations

#### 2.3.1. Solid Content

The determination of the solid content was as follows:

(1) $$\mathrm{Solid}\%=\left[\frac{W_{f}}{W_{s}}\right]$$

where *W_f_* and *W_s_* were the weights of dried emulsion and emulsion, respectively.

#### 2.3.2. Coagulum Content

The filterable solids were dried. The coagulum content was then calculated according to Equation (2):

(2) $$\mathrm{Coagulation}\%=\left[\frac{M_{f}}{solid\%\times M_{s}+M_{f}}\right]$$

where *M_f_* and *M_s_* were the weights of dried filterable solids and filtered latex, respectively.

#### 2.3.3. Viscosity

Viscosity was measured with a DV-79 digital viscometer (Worner Lab, Shanghai, China), employing E (10-100 mPa∙s) or F (100-1000 mPa∙s) rotor rotating at a velocity of 750 rpm at 25 °C.

#### 2.3.4. Particle Size

A small amount of emulsion was diluted to 1% with distilled water, and the particle size and particle size distribution of emulsion was determined by using a Zetasizer Nano ZS laser particle sizer (Malvern Instruments Ltd, Shanghai, China) at 25 °C.

#### 2.3.5. Transmission Electron Microscopy

Micrographs of the polyurethane–acrylate latex nanoparticles were obtained by JEM-2100 (JEOL Ltd., Tokyo, Japan) transmission electron microscope at an acceleration voltage of 200 kV. The emulsion was thinned to the appropriate concentration. Samples were stained with 3% phosphotungstic acid (PTA) solution. 

#### 2.3.6. Fourier Transform Infrared Spectroscopy

The waterborne emulsion was dropped on the potassium bromide tablets and dried under an infrared lamp, then put into a Nicolet-Nexus 670 FTIR (Thermo Fisher, Waltham, MA, USA) for analysis in the range of 4000 to 400 cm^−1^.

#### 2.3.7. Stability of the Emulsion

The emulsion was stored at room temperature for six months, or added to a CaCl_2_ solution or diluted with distilled water, and then observed whether there was a phenomenon of stratification, precipitation, flocculation, and so on, to evaluate its storage stability, chemical stability, and dilution stability, respectively.

#### 2.3.8. Preparation of Films

The emulsion was poured onto a polytetrafluoroethylene plate and dried for 24 h at room temperature, then dried at 60 °C for 48 h to obtain the film.

#### 2.3.9. Gel Permeation Chromatography

The number and average molecular weights were determined by gel permeation chromatography with tetrahydrofuran (THF) as the eluent, and polystyrene standards, as calibrated.

#### 2.3.10. Differential Scanning Calorimetry Measurements

The glass transition temperature (*T*_g_) of the polymer was measured in the range of −100 to 200 °C with a Mettler 822e differential scanning calorimeter under a nitrogen atmosphere at a heating rate of 10 °C/min.

#### 2.3.11. Thermogravimetric Analyzer

The heat resistance of the samples was tested on a Setsys16 thermogravimetric analyzer from Setrama, France. The sample weighed about 5 mg, in a nitrogen atmosphere, the heating rate was 10 °C/min, with a heating range of 20–600 °C. This method was used to test the thermal stability of the sample.

## 3. Results and Discussion

### 3.1. Effect of HEMA Dosage

The functional monomer HEMA was introduced because its molecular chain had a reactive functional group –OH. During the process of film formation, besides the multiple non-covalent interactions, such as hydrophobic interaction, hydrogen bonding, etc., the hydroxyl group can induce covalent crosslinking in high temperature [21]. The crosslinking density was increased by the introduction of the hydroxyl group, thereby improving the performance of the coating film. HEMA was used as the hydroxyl monomer, the total monomer amount was controlled, and the other monomer ratios were unchanged. Composite emulsions with different hydroxyl values were synthesized to study the effect of the hydroxyl content on the emulsion stability.

The effect of the amount of HEMA on the stability of the emulsion was shown in Table 1. When the amount of HEMA was 40%, the emulsion was completely gelled. The viscosity of emulsion increased with the increase of the amount of HEMA. After storage for 3 months at room temperature, 10% and 20% HEMA emulsions were layered, and 25%, 30% and 35% amount of HEMA emulsions were stable. The effect of hydroxyl monomers on emulsion polymerization stability can be considered from two perspectives: On the one hand, HEMA was extremely hydrophilic. When the content of water-soluble carboxyl monomers AA and MAA was constant, the hydrophilicity of the polymer was mainly determined by the content of surface hydroxyl groups. When HEMA was less than 20%, the polymer was phase-separated due to insufficient hydrophilicity. When the content of HEMA was more than 30%, the water-soluble monomer was too much, and it was easy to nucleate in the aqueous phase. When the macromolecular chain in the aqueous phase grew to a certain extent, it precipitated from the aqueous phase and became a gel [22]. On the other hand, when the content of hydroxyl monomer increased, the surface hydroxyl groups of acrylate latex particles increased, which made the number of hydrogen bonds formed by the hydroxyl group increased, and the molecular chains were entangled and surrounded by hydrogen bonds, resulting in the increase of emulsion viscosity, which affected the diffusion of the monomer in the emulsion system and the conduction of heat during the reaction, increased the cohesion rate, and reduced the stability of the polymerization reaction [23].

When the HEMA content was 25%~35%, the PdI showed that the dispersion of emulsion was moderate. The content of hydroxyl monomer should be increased as far as possible in order to ensure a certain crosslinking density under the condition of stable emulsion. Therefore, the amount of HEMA was set to be 35% of the total amount of acrylate monomers.

### 3.2. Effect of PET/AC Ratio

**Figure 2:**
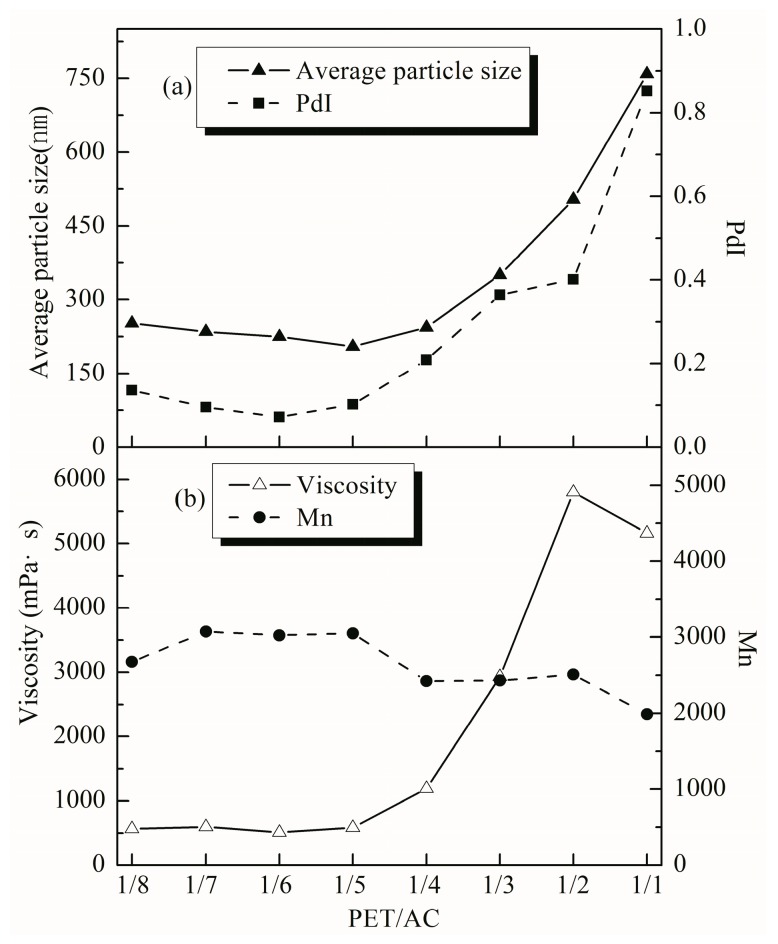
Effect of the PET/AC ratio on the properties of the emulsion. (**a**) Average particle size and PdI; (**b**) viscosity and *M*_n_. Conditions: The amount of HEMA was 30% of total weight of the PET and acrylate monomers, the ratio of AA/MAA/BA/St was 1.2/2.6/10.9/24.5 in this series reaction.

The effect of PET/AC ratio on the particle size and distribution of the emulsion is shown in Figure 2a. When the ratio of PET/AC was less than 1/4, the particle size and PdI of the emulsion was not much different. When the ratio of PET/AC was more than 1/4, the particle size of latex particles increased and the distribution became wider with the increased polyester dosage. The effect of PET/AC ratio on the viscosity and molecular weight of the emulsion was shown in Figure 2b. When PET/AC ratio was 1/5 to 1/8, the viscosity was not changed much and neither of them exceeded 1000 mPa∙s; when the PET/AC ratio was more than 1/4, the viscosity of emulsion increased obviously with the increased polyester dosage. When the PET/AC ratio was 1/2 and 1/1, the polyester content was high, and the viscosity of the emulsion was too large to flow. However, the amount of polyester had little effect on *M*_n_. When the ratio of PET/AC changed, the average molecular weight of PET-AC was basically in the range of 2000–3000. This is because the polyester was introduced into the acrylate macromolecular chain as a soft segment, the unsaturated bond in the polyester reacted with the acrylate monomer as the main chain, and the long molecular chain of the polyester became the side chain of the PET-AC, the steric hindrance was increased [24].

**Figure 3:**
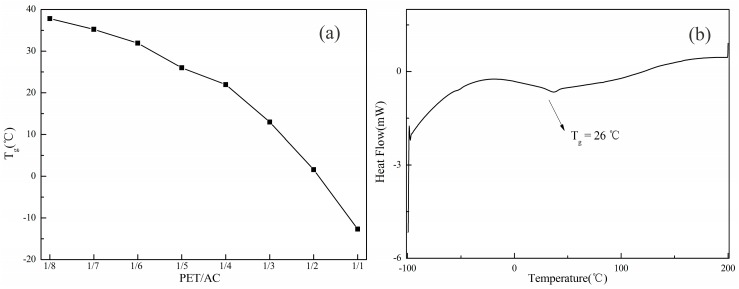
(**a**) Effect of the PET/AC ratio on the glass transition temperature of the film. (**b**) DSC thermogram of PET/AC was 1/5.

When the total monomer amount was constant, the amount of polyester was changed, the amount of HEMA was 30% of total weight of the PET and acrylate monomers, the proportion of AA/MAA/BA/St was controlled, and the amount of BMA was adjusted. The acrylic ester of each monomer homopolymer *T*_g_ is shown in Table 2, also as a soft monomer, the *T*_g_ of PET was −58 °C. Because the *T*_g_ of PET is similar to *T*_g_ of BA, they can be used as soft segments to make the film more flexible. In addition, the long molecular chain of the polyester has other effects on the polymer. When the ratio of PET/AC was changed from 1:1 to 1:8, the *T*_g_ ws tested by DSC, as shown in Figure 3a. The introduction of polyester reduced the *T*_g_ of the acrylate resin. When the rate of PET/AC changed from 1:1 to 1:8, with fewer dosages of polyester, the glass transition temperature of the resin increased from −12.67 to 37.8 °C. As a thermoplastic acrylate resin for the outdoor was used, *T*_g_ should be higher than the outdoor ambient temperature, about 25~40 °C, to prevent the resin from sticking back during high temperature in the summer [25].

When PET/AC was 1:5, DSC was used to test the thermal transformation of the PET-AC film, and the DSC curve is shown in Figure 3b. The glass transition temperature was only at 26 °C from the range of −100 to 200 °C, and there was no glass transition of polyester resin at −58 °C. It can be concluded that polyester with a double bond at the end group had been randomly copolymerized with acrylate and the compatibility was good.

**Figure 4:**
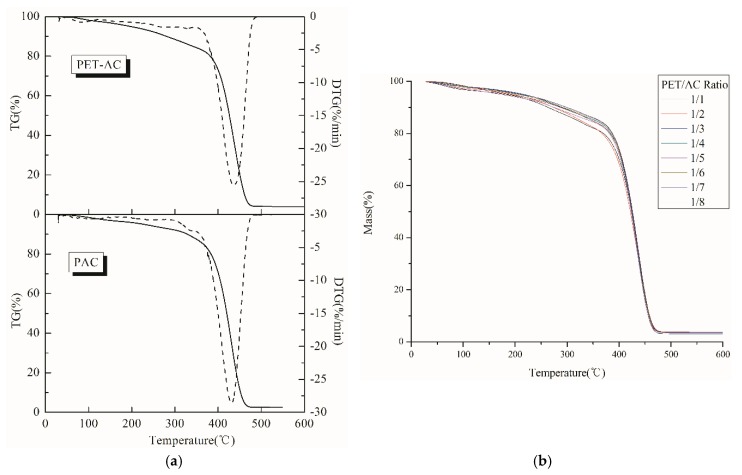
(**a**) TGA/DTGA curves of PET-AC and PAC; (**b**) Thermogravimetric analysis of different ratios of PET/AC.

In order to investigate the effect of PET/AC ratio on the thermal stability of resins, the thermogravimetric curves of emulsion with different PET/AC ratio were determined. It is shown in Figure 4a that with the introduction of polyester, the initial decomposition temperature decreased obviously and the maximum weight loss temperature of the film remained nearly unchanged. From Figure 4b, the initial weight loss temperatures of films that the PET/AC ratio of 1/1 and 1/2 were significantly lower than other ratios, indicated that the initial thermal decomposition temperature of the composite resin decreased significantly with the addition of a large number of polyesters. Considering the comprehensive performance of the emulsion, the ratio of PET/AC was defined as 1/5 in this paper. 

### 3.3. Effect of Soft/Hard Monomer Ratio

The acrylate monomers can be classified into two types depending on the glass transition temperature of the homopolymer. The monomers having a higher *T*_g_ are a hard monomer which impart hardness and abrasion resistance to the latex film and provides adhesion. Soft monomers have a lower *T*_g_ that provide flexibility and viscosity to the latex film. The effect of the ratio of soft monomer BA to hard monomer St on the emulsion properties was investigated under the condition that the other monomers were kept unchanged.

**Figure 5:**
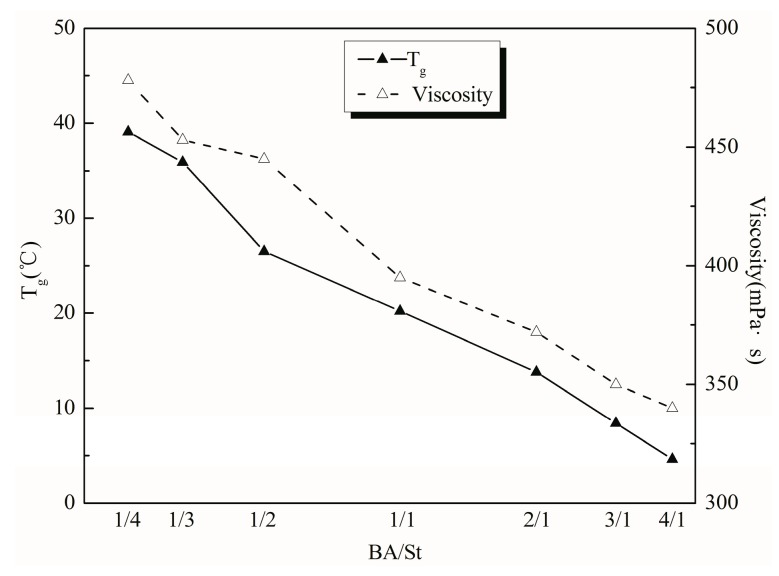
Effect of soft/hard monomer ratio on *T*_g_ and viscosity. Conditions: The ratio of AA/MAA/HEMA/BMA was 1.2/2.6/35/40.7 in this series reaction.

As can be seen from Figure 5, the viscosity of the emulsion increased as the content of the hard monomer St increased. When the proportion of BA was less than St, the hard monomer accounted for a larger component. There were many active side groups on the molecular chain of resin, and the molecular chain was hindered by steric hindrance, which made it difficult to move. This increased more hydrogen bonds among the molecules, which led to a higher viscosity of emulsion. When there is more BA in the system and St is less, the soft monomer accounts for larger components, and the molecular chain is more flexible. The steric hindrance of the movement was smaller, the polymer chain was curled, and the viscosity was lowered. The *T*_g_ of the polymer decreased as the soft monomer BA content increased. When the *T*_g_ was too low, the emulsion would become sticky after film formation, and when the *T*_g_ was too high, the emulsion would become hard and brittle after film formation, which resulted in poor flexibility [26]. Considering the factors above, the suitable soft/hard monomer ratio was 1/3.

### 3.4. Effect of Chain Transfer Agent Content

The chain transfer agent regulates the molecular weight of the polymer by a transfer reaction to the chain radical and tends to narrow the distribution of the relative molecular mass. The average molecular weight of the high solids coating is generally from 2000 to 10,000 [27]. The polymerization activity of the acrylate monomer is large, and the emulsion polymerization tends to cause the molecular weight of the copolymer to be large, so that the viscosity of the resin is large, so that the coating performance of the coating is affected. Therefore, it is often necessary to add an appropriate amount of chain transfer agent in the polymerization process to reduce the relative molecular mass, reduce the viscosity, and meet the application and construction requirements of the coating. However, in terms of resin structure, when the relative molecular mass of the resin is reduced to a certain extent, it is difficult to ensure that there are more than two hydroxyl groups per molecular chain of the resin, so that it cannot crosslink well with the curing agent to form a bulk macromolecule, which reduces the performance of coating.

**Figure 6:**
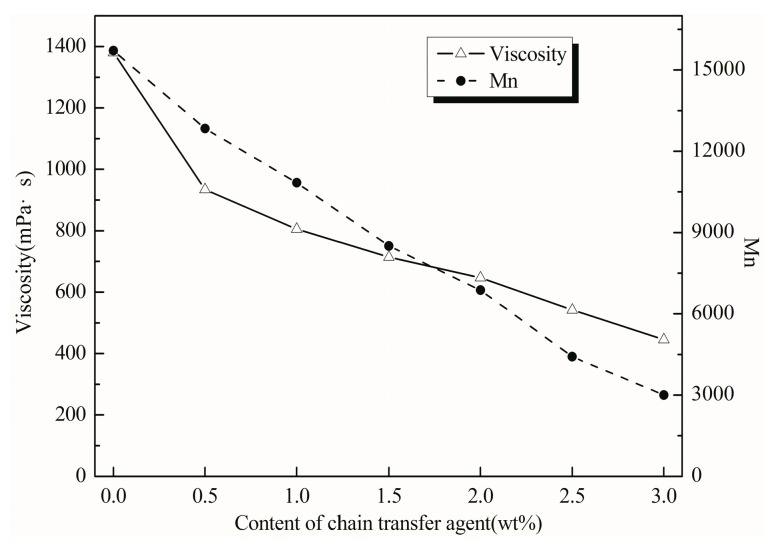
Effect of the content of chain transfer agent on viscosity and molecular weight. Conditions: The ratio of PET/AC was 1/5, and AA/MAA/BA/St/HEMA/BMA was 1.2/2.6/5.2/15.3/35/40.7 in this series reaction.

It can be seen from Figure 6 that the change trend of the viscosity and molecular weight of the resin was consistent when the amount of the chain transfer agent was changed. As the amount of 1-dodecanethiol increased, the viscosity and molecular weight of the resin decreased. Since the chain transfer agent concentration was increased, the chain termination rate was increased, and the relative molecular mass of the resin was lowered, so the viscosity was lowered. However, when the concentration of the chain transfer agent was increased, the amount of the initiator to be consumed was increased, and the efficiency for actually initiating the reaction was lowered, so that the conversion rate of the monomer was lowered, and the coating film performance was deteriorated. The viscosity of the emulsion was too high, which was not conducive to the construction and flow-levelling [28]. When the viscosity of the emulsion was too low, the storage stability and sag resistance of the emulsion would deteriorate [29]. Considering the factors above, the content of the chain transfer agent was 1.0%.

### 3.5. FTIR Spectra

**Figure 7:**
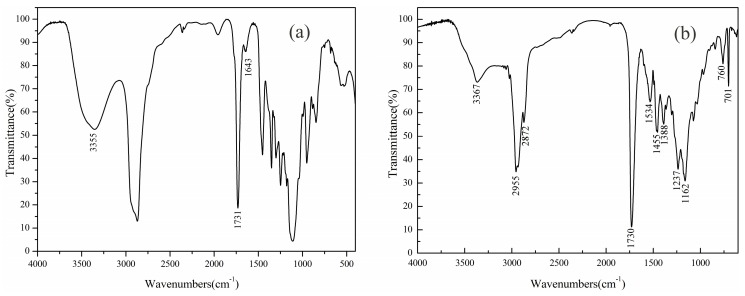
FTIR spectra of (**a**) PET and (**b**) PET-AC emulsion.

According to Figure 7a, the absorption peak at 1730 cm^−1^ was the stretching vibration peak of C=O, and the peak at 3355 cm^−1^ was the O–H stretching vibration absorption peak, which indicated that polyester reacted with hexahydrophthalic anhydride to introduce carboxyl groups. The peak at 1643 cm^−1^ was the characteristic peak of C=C. It can be seen that double bonds were introduced by the transesterification reaction between polyester and MMA. As can be seen from Figure 7b, the broad peak at 3367 cm^−1^ was the O-H stretching vibration absorption peak of hydroxyl functionalized acrylate. Two peaks at 2872 and 2955 cm^−1^ were ascribed to the C-H stretching vibrations of -CH_2_ and –CH_3_ groups, respectively, while the bending C–H vibrations of –CH_2_ and –CH_3_ can be found at 1455 and 1388 cm^−1^, respectively. The strong absorption peak at 1730 cm^−1^ was the stretching vibration peak of C=O, and the intensity was higher. 760 and 701 cm^−1^ were characteristic absorption peaks of a single substituted benzene ring. The band at 1160 cm^−1^ were stretching vibrations of the C–O–C group. The absorption peak of 1237 cm^−1^ belonged to RO–SO^3−^ and C–O–C and SE-10 was involved in the polymerization of the emulsifier. In addition, the characteristic absorption peak of C=C at 1600 cm^−1^ completely disappeared, indicating that the monomers were reacted. The waterborne PET-AC emulsion was successfully prepared by emulsion polymerization.

### 3.6. Comparison of PAC and PET-AC

**Figure 8:**
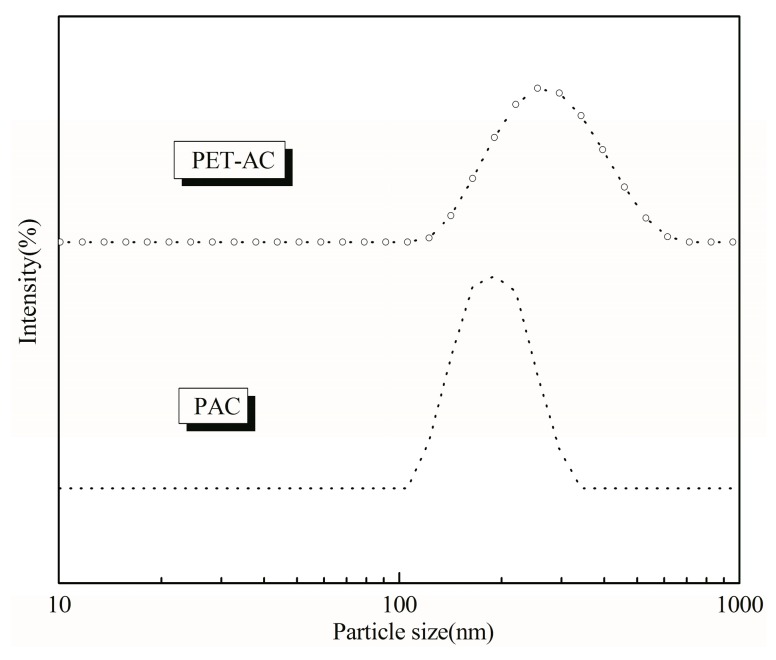
Particle size distribution of latex particles before and after polyester modification.

**Figure 9:**
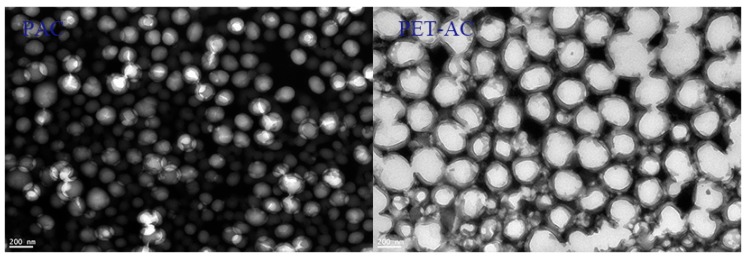
TEM micrograph of the PAC and PET-AC latex particles.

According to Table 3, the solid content of emulsion after polyester modification was slightly higher than the pure PAC. From Figure 8, the PET/AC emulsion had a larger average particle size and wider distribution than PAC emulsion. As can be seen from Figure 9, the particle size of PET-AC latex particles was obviously larger than that of PAC, and the arrangement was more compact. According to the mathematical model of random compact accumulation of spherical objects [30,31], the wider the size distribution, the greater the packing density. Therefore, the solid content of PET/AC emulsion was higher than PAC emulsion [32,33]. The viscosity of PET/AC was greater than PAC, which made it better in sag resistance.

Coatings used for automobiles were prepared with PET-AC resin as the main film forming materials and amino resin as the curing agent. The performance of the film was determined according to the relevant standards. Table 4 shows that the hardness of the emulsion after film formation was slightly reduced with the addition of polyester, but the flexibility, adhesion and impact resistance were superior to those before modification. In summary, polyester modified hydroxyl polyacrylate emulsion had good performance and application prospects.

## 4. Conclusions

In this paper, polyester resin was introduced to modify the hydroxyl acrylate emulsion in order to solve the problem of low flexibility, abrasion resistance and poor impact resistance. The carboxyl terminated unsaturated polyester was first synthesized and added as a monomer to copolymerize with the acrylate monomer, and the polyester modified hydroxyl acrylate emulsion was prepared by a seed pre-emulsification semi-continuous emulsion polymerization method. When the dosage of HEMA was 35% of the total amount of acrylate monomers, the ratio of PET/AC was 1/5, the ratio of soft/hard monomer was 1/3 and the content of chain transfer agent was 1%, the waterborne polyester-acrylic composite emulsion could be prepared. Through a variety of tests, we proved that the waterborne polyester-acrylate composite emulsion was successfully prepared with high hydroxyl content, high solid content, good stability, good sag resistance and good mechanical properties. The coatings prepared from polyester modified hydroxyl polyacrylate had good film properties and could be used in the automobile industry. Therefore, the environmental-friendly polyacrylate emulsion will replace the traditional solvent based coatings and will be widely applied in the coatings industry.

## Figures and Tables

**Table 1:** Effect of HEMA dosage on the properties of emulsion.

HEMA (wt %)	Viscosity (mPa*s)	Particle Sizer (nm)	PDI	Storage Stability	Solid (%)
10	-	-	-	Demulsification	-
20	-	-	-	Demulsification	-
25	71	313	0.141	Stable	42.5
30	281	266	0.158	Stable	46.0
35	598	204	0.102	Stable	48.0
40	613	-	-	All gels	45.0

Conditions: PET/AC was 1/5, and the ratio of AA/MAA/BA/St was 1.2/2.6/10.9/24.5 in this series reaction.

**Table 2:** The glass transition temperature of the monomer.

Monomer	PET	AA	MAA	BA	St	HEMA	BMA
*T*_g_ (°C)	−58	106	130	−56	100	55	21

**Table 3:** Comparison of PAC and PET-AC on the properties of emulsion.

	Solid (%)	Particle Sizer (nm)	PdI	Viscosity/mPa·s	*M*_n_
PAC	46.5	156.4	0.067	314	10,570
PET-AC	50.0	255.6	0.110	805	10,850

Conditions: The ratio of PET/AC was 1/5, AA/MAA/BA/St/HEMA/BMA was 1.2/2.6/5.2/15.3/35/40.7, and the content of 1-dodecanethiol was 1%.

**Table 4:** The properties of aminoacrylic coating film.

Properties	PAC	PET-AC
Film appearance	Smooth and flat	Smooth and flat
Water resistance ^a^	Pass	Pass
Pendulum hardness ^b^	100 s	90 s
Impact strength ^c^	30 kg·cm	50 kg·cm
Adhesion ^d^	1 degree	0 degree
Flexibility ^e^	2 mm	1 mm

^a, b, c, d, e^: Determined by the method in GB/T 1733-93, 1730-93, 1732-79, 1720-79, 1731-93 respectively.
